# Evaluation of Lipid-based Drug Delivery System (Phytosolve) on Oral Bioavailability of Dibudipine

**Published:** 2014

**Authors:** Fariborz Keyhanfar, Samira Khani, Shahab Bohlooli

**Affiliations:** a *Pharmacology Department, Iran University of Medical Sciences, Tehran, Iran.*; b *School of Pharmacy, Ardabil University of Medical Sciences, Ardabil, Iran. *

**Keywords:** Dibudipine, Phytosolve, Bioavailability, Solubility, Lipid-based formulation

## Abstract

The objective of present study was to evaluate pharmacokinetic parameters of dibudipine Phytosolve after oral administration in rats. The solubility test was carried out to select suitable oily solvent for dibudipine. Phytosolve formulation was prepared with a medium chain triglyceride (MCT) oil (20%), soybean phospholipids (5%) and a 70% fructose solution (75%). The effect of polyol content on the mean globule size of Phytosolve formulation was studied. The optimized formulation was evaluated for robustness toward dilution, transparency, droplet size, zeta potential and transmission electron microscopic analysis. The Phytosolve of dibudipine with an average droplet size of 142.3 ± 4.3 nm and surface charge -18.36 ± 0.37 mv was administered orally to rats. The average relative bioavalabilities of dibudipine in the plasma with Phytosolve were 170.4% and 211.2% as compared to the oily solution and aqueous suspension respectively. So this formulation could be offered as a useful technique to improve the oral delivery of the poorly water soluble drugs such as dibudipine.

## Introduction

Dibudipine (bis-t-butyl, -1, 4-dihydro-2, 6-dimethyl-4 -(3-nitrophenyl)-3, 5-pyridine dicarboxylate) is a new calcium channel blocker which synthesized by Mahmoudian *et al*. in 1997 (1). It is a potent vasodilator with antihypertensive activity (2). Dibudipine has longer half-life (2.5 h) than other dihydropyridine (DHP) derivatives such as nitrendipine, nicardipine, felodipine, nislodipine and nifedipine (3). 

However pharmacokinetic studies have indicated very low oral bioavailability and high inter and intra subject variation in plasma concentration of dibudipine (4). It is likely due to low water solubility and high first pass effect the same as other DHPs such as lacidipine (5) and nitrendipine (6). 

In order to enhance clinical efficacy of this molecule, it is important to develop a new formulation to overcome these problems. In recent years lipid-based formulations have attracted researcher’s attention to themselves (7-8). Many studies have shown that these formulations could improve the oral bioavailability and plasma concentration profile of drugs with low water solubility (9). 

Phytosolve is a novel solubilizing technique which introduced by Wajda R. in 2003. In this technique phospholipids, dispersed in highly concentrated aqueous solution of polyol or carbohydrate, are able to solubilize large quantities of lipids, steroids, terpenes and polar lipids(10). The present study aimed at developing an optimal Phytosolve of dibudipine and evaluating its pharmacokinetic potential.

## Experimental

Dibudipine and mebudipine were purchased from Pars Biopharmacy Research Co. (Tehran, Iran). Medium chain triglyceride (MCT), soybean phosphatidylcholine (Lipoid S75) kindly provided by Lipoid GmbH (Ludwigshafen, Germany). HPLC grade acetonitrile, methanol and dichloromethane were obtained from Merck (Darmstadt, Germany). Double distilled water was obtained through a Milli-Q system (Millipore, USA). The vegetable oils were provided by Barij Essence Co. (Kashan, Iran).

*Solubility study*

The solubility of dibudipine in different oils including MCT, Ethyl oleate, Thistle oil, Olive oil, Almond oil, Walnut oil, Flax seed oil and Mineral oil was determined by saturation solubility technique (11). An excess amount of the dibudipine added to small volume (1 mL) of the solvent and vortexed for 10 min. After equilibration by shaking at room temperature for 48 h, the mixture was centrifuged at 3000rpm for 20 min. The supernatant was withdrawn and filtered through a 0.2 μm syringe filter then analyzed by high performance liquid chromatography (HPLC).

*Preparation of dibudipine* *phytosolve*

For preparation of Phytosolve formulation, 20 mg of dibudipine was dissolved in 2 g of MCT oil. 0.5 g of phospholipid (Lipoid S75) was dispersed in the 7.5 g of polyol phase at room temperature and homogenized using an Ultra-Turax homogenizer (IKA T10B, Germany). The oily phase (MCT plus dibudipine) was added slowly to the phospholipid-polyol phase and mixed well. Final mixture was sonicated with a probe sonicator (Hielscher, Germany) with a 70% amplitude and cycle 0.6 for 10 min to decrease particle size in emulsion. Different Phytosolve formulations were prepared with various polyol phases such as glycerol, a 70% fructose solution or a 70% sucrose solution to achieve an optimum formulation. 

*Characterization of formulation*

*Measurement of size and zeta potential*

The mean particle size, poly dispersity index and zeta potential of the Phytosolve formulation were measured using photon correlation spectroscopy instrument, zetasizer Nano (Malvern, UK) at 25 °C. The formulation was diluted with distilled water (1:100) to avoid particle interaction during measurement.

*Physical robustness to dilution*

The formulations were diluted 50, 100, 500 and 1000 times with double distilled water. The diluted emulsion were evaluated for any physical changes such as precipitation or phase separation after 24 h, one week and one month storage in 4 °C.

*Percentage transmittance*

Phytosolve formulations were diluted 200 times with distilled water and %Transmittance was measured using UV-Visible spectrophotometer (Pharmacia Biotech, UK) at 650 nm against distilled water as blank. The stability of different Phytosolve formulations (DPF, DPG, DPS) were assessed through transmittance measurement 30 minute after preparation and 7 days later.

*Transmission electron microscopy*

A drop of diluted formulation were deposited on a 200 mesh holey film grid and stained with uranyl acetate after drying. The samples were observed with LEO 906 transmission electron microscope (ZEISS, Germany).

*Pharmacokinetic study*

Male Wistar rate weighing 250-300 mg were obtained from the Razi institute (Karaj-Iran). The protocol of animal experimental study was approved by ethics committee of the Tehran University of Medical Sciences. The animals were kept in standard condition (25 ± 2^°^ C and 55 ± 5% humidity). They fasted overnight with free access to water before drug administration. The Phytosolve and control formulations (oily solution, suspension) were administered orally (10 mg/Kg) with a gavage needle. The dibudipine suspension was prepared with hydroxymethylcellulose as suspending agent in water. The blood samples (500 μL) were collected via a catheter which implanted 2 days before the experiment in the jugular vein according to Thrivikraman reported protocol (12) in heparinized tube at 0, 10, 20, 30, 60, 90, 120, 240 and 360 min after orally administration. The plasma was separated by centrifugation at 5000 rpm for 20 min and stored at -20^ ͦ^ C until analysis with HPLC.

*Analysis of dibudipine concentration*

A reverse phase HPLC system containing a 600 pump, a UV-vis detector and manual injector (Youngling, Korea), software (Autochro-2000) and a tracer excel ODS-A analytical column (4.6*250 mm, 5 um) was used to assay the plasma concentration of dibudipine. The mobile phase was methanol:acetonitrile:water (75:5:25) with flow rate 1 mL/min. The UV detector was adjusted on 238 nm for analysis.

*Plasma sample preparation*

Mebudipine was used as internal standard and 10 μL (4 μg/mL) of it was added to 200 μL of the plasma sample. There were mixed using a vortex mixer and 200 μL of NaOH (1 N) was added to them. After mixing for 1 min, 2 mL of dichloromethane was added and vortexed vigorously for 5 min and centrifuged at 5000 rpm for 25 min. The organic layer was transferred to a clean tube and evaporated at 40^ °^C under nitrogen flow. The residue was reconstituted in 100 μL of the mobile phase and the solution (20 μL) was injected on to the HPLC for analysis.

 The calibration curve for linearity was plotted using five concentrations (10, 50, 100, 500 and 1000 ng/mL) of dibudipine in blank plasma. The recovery percentage of dibudipine was determined by comparing the peak area of the extracted dibudipine from the plasma with the peak area of obtained by the direct injection of a pure standard dibudipine in mobile phase at three different concentrations (100, 500 and 1000 ng/mL).

The accuracy and precision of method and interday, intraday analysis variability were determined over the four concentrations (10, 100, 500 and 1000 ng/mL) in the blank plasma. 

Various pharmacokinetic parameters (Cmax, Tmax, AUC and T_½_) were calculated for dibudipine Phytosolve, oily solution and suspension. The percent relative bioavailability (%F) of Phytosolve to reference formulations (suspension and oily solution) was calculated as bellow:

%F = (AUC_0-__∞_ product/AUC_0-__∞_ reference) ×100 

The maximum concentration (Cmax) and the time needed to reach the maximum concentration (Tmax) values were obtained directly from the concentration versus time curve.

The area under the plasma drug concentration-time curve from 0 to 6 h (AUC_0-6_) was calculated through the trapezoidal method. The area under the plasma drug concentration-time curve from 0 to infinity (AUC_0-∞_) was calculated via the sum of the areas obtained by the trapezoidal method (AUC_0-6_) and residual area (AUC_6-∞_). The residual area and T_½_ were obtained according to following equations (13).

AUC_t-∞_ = Ct/k_e_

 T_½_ = ln2/k_e_

*Statistical analysis*

All data are reported as mean ± S.D. Statistical comparisons were made using one way analysis of variance (ANOVA) followed by Tukey post hoc test at a level of significance of P < 0.05 using SPSS software (version 14).

## Results and Discussion

*Solubility study*

**Figure1:**
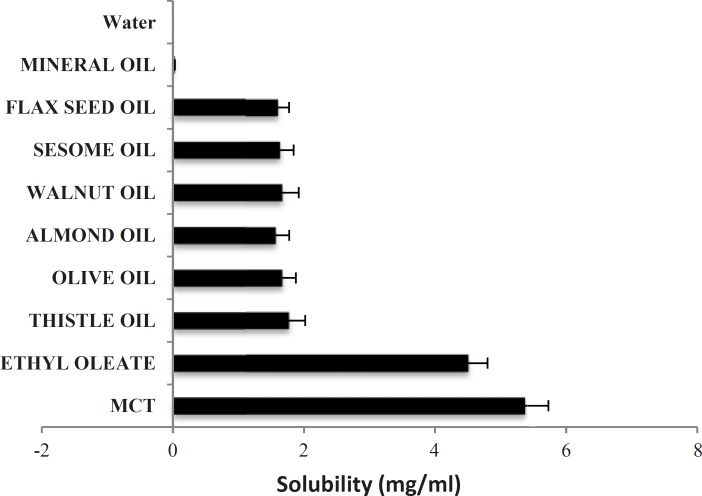
Solubility of dibudipine in various oils; data expressed as the mean ± S.D (n=3).

To find out an appropriate oily phase in Phytosolve formulation, the solubility of dibudipine in various oils was tested. The solubility of dibudipine was found to be higher in MCT than other oils (Figure 1) so this oil was selected as the solvent. 

In the present study the effect of a new lipid-based formulation (Phytosolve) on pharmacokinetic parameters of dibudipine after oral administration in rats was evaluated. Dibudipine belongs to 1, 4- dihydropyridines class and has low water solubility. The solubility of dibudipine in water is 0.00043 mg/mL. In order to select suitable oil with good solubilizing capacity for dibudipine, the solubility study was done. The results have shown MCT is better solvent respect to the other oils. Previous studies indicated MCTs were used in many formulations due to high fluidity, good solubility properties and emulsification ability (14), promoting bioavailability and lack of pharmacological action (15).

*Selection of polyol phase*

The polyol phase is another component in the Phytosolve formulations. It decreases polarity of continuous phase and interfacial tension (16) and facilitates microemulsification of formulations (17). In order to evaluate the effect of polyol phase on the particle sizes of formulations, different formulations were analyzed using zetasizer. The results (Table 1) have shown the kind of polyol has not any significant effect on the particle size and zeta potential of formulations. 

**Table 1:** Composition, mean droplet size, polydispersity index and zeta potential of different Phytosolve formulations.

**Code**	**Percentage w/w of different components in formulation**			**Mean droplet size ±S.D. (nm)**	**PDI±S.D.**	**Mean zeta potential ±S.D.(mv)**
	Oil(20%)	Polyol(75%)	Phospholipid(5%)			
DPF	MCT	Fructose	Lipoid S75	142.3 ± 4.3	0.12 ± 0.02	-18.36 ± 0.37
DPG	MCT	Glycerol	Lipoid S75	147.3 ± 6.1	0.12 ± 0.01	-18.29 ± 0.16
DPS	MCT	Sucrose	Lipoid S75	148.5 ± 10.9	0.11 ± 0.02	-18.49 ± 0.10

PDI indicates polydispersity index.

DPF, DPG and DPS: dibudipine Phytosolve formulation which contained fructose solution, glycerol and sucrose solution as polyol phase respectively.

Dibudipine was dissolved in MCT (10 mg/g) in all the formulation (DPF, DPG, DPS).

The percentage transmittance of the Phytosolve formulations (DPF, DPG, DPS) is indicated in Table 2. It can be seen from the table that the formulation DPF shows a very low difference in transmittance during 7 days as compared to other formulations. A lower value of transmittance differenc in formulation DPF can indicate the stability of this formulation.

**Table 2:** The percent transmittance of dibudipine Phytosolve formulations at 630 nm

Phytosolve Code	%Trancmittance after 30 min	%Transmittance after 7 days	Transmittance difference
DPF	69.7 ± 7.6	65.4 ± 9.2	4.3
DPS	61.0 ± 9.0	69.3 ± 10.0	-8.3
DPG	58.6 ± 9.5	50.3 ± 10.5	8.3

Data expressed as the mean ± S.D (n=3).

DPF, DPS and DPG: dibudipine Phytosolve formulation which contained fructose solution, sucrose solution and glycerol as polyol phase respectively.

*Characterization of selected Phytosolve formulation*

The main property of this formulation is the use of natural emulsifiers. The ionic and nonionic surfactants with short chain alcohol or amine cosurfactant were used in many formulations to form stable microemulsions. However the high toxicity of these surfactants limited their pharmaceutical usage and biocompatible zwitterionic naturally obtained surfactant such as lecithin were replaced with them in the new formulations (18).

The Phytosolve formulation containing fructose (DPF) which indicated smaller particle size and lower change in transmittance during storage was selected for the pharmacokinetic experiments. The selected formulation (DFP) was exposed to different fold of dilution (50, 100, 500 and 1000 times) with double distilled water. The results (no sign of separation and precipitation) demonstrated this formulation was able to mix with water in any ratio. The Phytosolve formulation was robust to dilution so it is expected that after dilution in Gastrointestinal (GI) fluid, dibudipine precipitation will not be occurred. 

 On the other hand the formulation had small particle size (142.3 ± 4.3 nm) and poly dispersity index (0.12 ± 0.02) which reflects the uniformity of particle diameter. In order to obtain small particle size, high energy input was needed. A probe sonicator was used in this study instead of high pressure homogenizer which used before. So the desired particle size (30-60 nm) was not achieved (10). 

The zeta potential measurement is used to identify the surface charge of droplets. The zeta potential of Phytosolve was -18.36 ± 0.37 mv. The percentage transmittance of the optimized formulations was 69.7 ± 7.6. As shown in Figure 3, the morphology of droplets was spherical and their size was similar to the results obtained using zetasizer.

**Figure 2:**
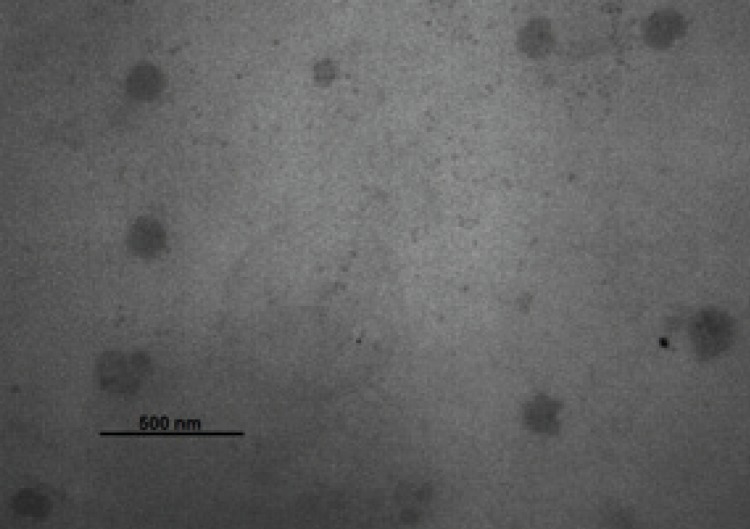
Transmission electron micrographs of the Dibudipine-containing Phytosolve. The scale bar represents a distance of 500 nm

*HPLC analysis*

**Figure 3:**
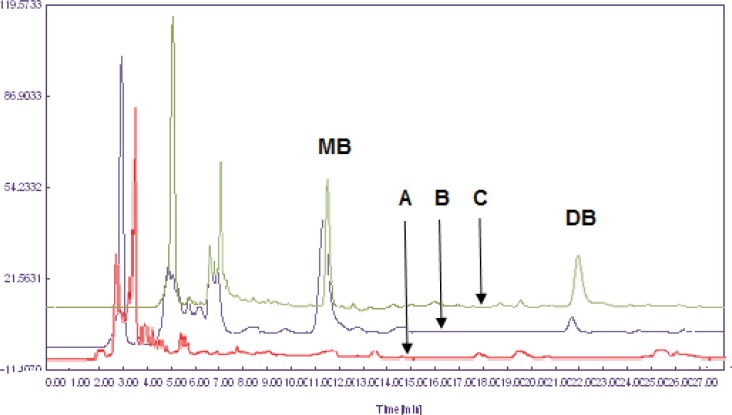
Overlay chromatograms of (A) blank rat plasma, (B) rat plasma spiked with dibudipine (LLOQ) and IS (400 ng/mL) and (C) rat plasma sample after oral administration of 10 mg/Kg dibudipine .

The extraction of dibudipine from the plasma samples was carried out using a liquid-liquid extraction procedure with three different solvents: ethyl acetate, ethyl ether and dichloromethane. Extraction recovery was higher with dichloromethane when compared with other organic solvents hence it was used as extraction solvent. Representative chromatograms of the blank plasma**,** a plasma spiked with 10 ng/mL (LLOQ) of dibudipine and 400 ng/mL of internal standard (mebudipine) and a plasma after oral administration of dibudipine are presented in Figure 3. No interfering peaks from endogenous compounds are observed at the retention times of analytes. Retention time of dibudipine was 22 min and for internal standard was 11.9 min.

The assay exhibited good linearity (r^2 ^=0.9954) over the range of 10-1000 ng/mL for dibudipine. The lower limit of quantification was 10 ng/mL which is acceptable for pharmacokinetic studies. The coefficient of variation and error were less than 10% for all of intraday and interday validation (Table 3). The recovery of dibudipine was found to be over 80% indicating the ability of dichloromethane in extraction of the drug from plasma. 

**Table 3:** The precision and accuracy of dibudipine determination in rat plasma

**Sample concentration (ng/mL)**	**10(LLOQ)** a	**100(low)**	**500(Medium)**	**1000(High)**
Intra-day(n=5)				
**Mean ± S.D.**	**9.2 ± 0.7**	**91.4 ± 4.1**	**450.0 ± 18.7**	**940.0 ± 41.8**
**CV (%)** b	**8.1**	**4.5**	**4.15**	**4.4**
**Error (%)** c	**-7.8**	**-7.6**	**-10.0**	**-6.0**
Inter-day(n=5)				
**Mean ± S.D.**	**9.1 ± 0.3**	**90.8 ± 2.9**	** 502.0 ± 37.0**	**920.0 ± 25.5**
**CV (%)**	**3.8**	**3.2**	** 7.3**	**2.7**
**Error (%)**	**-9.0**	**-9.2**	** 0.4**	**-8.0**

The measured concentration minus the expected concentration to the expected concentration × 100.

a LLOQ: lower limit of quantification

b Precision (at each concentration) was expressed as CV% = (SD/ mean measured concentration) × 100

c Accuracy (at each concentration) was expressed as the Error%, which was calculated by dividing

*Pharmacokinetic study*

**Figure 4:**
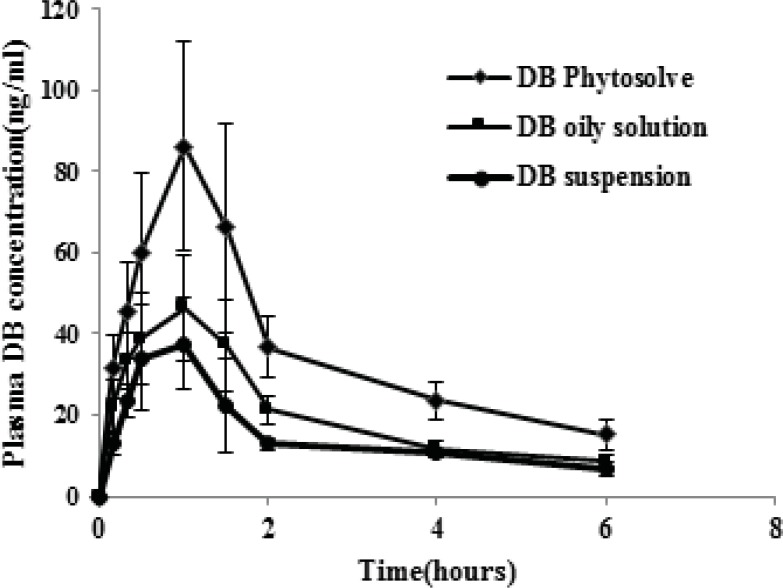
Drug-concentration time profiles of various dibudipine (DB) formulations after oral administration to rats. (n =6, dose=10 mg/Kg).

 The plasma concentration-time profiles of dibudipine after oral administration of Phytosolve, oily solution and suspension are illustrated in Figure 4. The pharmacokinetic parameters of dibudipine after oral administration of various formulations are summarized in Table 4. The Cmax, AUC_0-6 h_ and AUC_0-∞_ values of dibudipine Phytosolve were significantly (P < 0.001) higher than those obtained with oily solution and suspension but the Tmax was not significantly different between three formulations. Dibudipine Phytosolve provided mean relative bioavailabilities of 170.4% and 211.2% as compared to dibudipine oily solution and suspension respectively.

The *in-vivo* study has shown plasma concentration time profile of dibudipine Phytosolve represented greater improvement of dibudipine bioavailability than oily solution and suspension. The previous study was indicated fivefold increase in bioavailability of Co Q10 and 10-fold in the case of vitamin E after administration of Phytosolve formulation in healthy volunteers (19). It was mentioned that phospholipids can act as a sorption promoter (19). Another study also have reported phospholipids and particularly, phosphatidylcholine can enhance lymphatic transport of halofantrine (20). Furthermore phospholipids can protect drug from decomposition by enzymes and bacterial destruction in the GI tract (21). The particle size plays a dominant role in the absorption of drugs in the GI tract. A Phytosolve particle size of approximately 150 nm may allow for efficient uptake in the intestine, particularly in the lymphoid sections of this tissue and therefore escapes from the first pass metabolism in the liver. The presence of phospholipids in this formulation besides small particle size which provide large interfacial area for drug absorption may be the possible causes of improvement bioavailability of dibudipine by Phytosolve.

**Table 4:** Pharmacokinetic parameters upon oral administration of various dibudipine formulations.

Pharmacokinetic parameters	Suspension	Oily solution	Phytosolve
C_max_(ng/mL)	42.2 ± 7.2	60.1 ± 6.3	99.0 ± 19.1^a^^b^
T_max_(h)	1.00 ± 0.31	1.08 ± 0.20	1.16 ± 0.25
T_1/2_(h)	4.45 ±1.21	3.37 ± 1.08	3.21 ± 0.64
AUC_0→6 _(ng h/mL)	91.1 ± 9.5	126.7 ± 10.2	217.9 ± 33.4^a^^b^
AUC_0→∞ _(ng h/mL)	137.5 ± 29.5	170.4 ± 11.7	290.4 ±59.9^a^^b^

Data expressed as the mean±S.D., n=6

a Significantly higher (*p *< 0.001) compared to dibudipine suspension.

b Significantly higher (*p *< 0.001) compared to dibudipine oily solution

## Conclusion

The results of this study have shown Phytosolve formulation improved the oral bioavailability of dibudipine in rats and confirmed that Phytosolve could be used as an effective lipid-based drug delivery system for poorly water soluble drugs such as dibudipine.
